# Influence of RT-qPCR primer position on EGFR interference efficacy in lung cancer cells

**DOI:** 10.1186/1480-9222-13-1

**Published:** 2010-11-11

**Authors:** Gang Chen, Peter Kronenberger, Erik Teugels, Jacques De Grève

**Affiliations:** 1 Laboratory of Molecular Oncology and Department of Medical Oncology, Universitair Ziekenhuis Brussel, Vrije Universiteit Brussel, Brussels, Belgium; 2 Department of Pathology, First Affiliated Hospital, Guangxi Medical University, Nanning Guangxi, PR China; 3 Laboratory for Biotechnology, Department of Gezondheidszorg, Erasmushogeschool Brussel, Brussels, Belgium

## Abstract

**Background:**

Real-time quantitative RT-PCR (RT-qPCR) is a "gold" standard for measuring steady state mRNA levels in RNA interference assays. The knockdown of the epidermal growth factor receptor (EGFR) gene with eight individual EGFR small interfering RNAs (siRNAs) was estimated by RT-qPCR using three different RT-qPCR primer sets.

**Results:**

Our results indicate that accurate measurement of siRNA efficacy by RT-qPCR requires careful attention for the selection of the primers used to amplify the target EGFR mRNA.

**Conclusions:**

We conclude that when assessing siRNA efficacy with RT-qPCR, more than one primer set targeting different regions of the mRNA should be evaluated and at least one of these primer sets should amplify a region encompassing the siRNA recognition sequence.

## Background

RNA interference (RNAi) can mediate a short-term or prolonged silencing of gene expression at the RNA and protein level. Knockdown efficiency is typically measured at the mRNA level by quantitative RT-PCR (RT-qPCR) or estimated at the protein level by immunoblot, enzyme-linked immunosorbent assay (ELISA) or immunohistochemistry (IHC) [1]. Many prefer measuring the relevant protein with immunoblot directly, because protein knockdown is most relevant to the observable phenotype under study. However, in practice, a suitable antibody to a given target protein may not always be readily available or will not allow a quantitative estimate of the magnitude of the effect of RNAi. The long turnover time of many proteins may underestimate the RNAi effect at the mRNA level. A direct measurement at the mRNA level is therefore often the preferred method to more directly verify that RNAi is effectively decreasing the amount of the transcript. Real-time RT-qPCR is the "gold" standard for measuring steady-state mRNA levels. Hence, an accurate measurement method of the mRNA knockdown is needed. There are indications that mRNAs are not completely degraded after 24 hrs of RNAi exposure [2]. Therefore the location of the primer might be relevant as some primer sets may amplify remaining cleavage products, leading to an underestimation of the RNAi efficacy [3]. Despite this, numerous publications on RNAi with RT-qPCR do not evaluate the primers choice. The lack of precise criteria for choosing the target sequence for RT-qPCR amplification is surprising. Here, evidence is presented that the location of RT-qPCR primers is critical in the evaluation of the epidermal growth factor receptor (EGFR) small interfering RNA (siRNA) efficacy, even up to 72 hrs post-treatment in lung cancer cells.

## Results and Discussion

**Figure 1:**
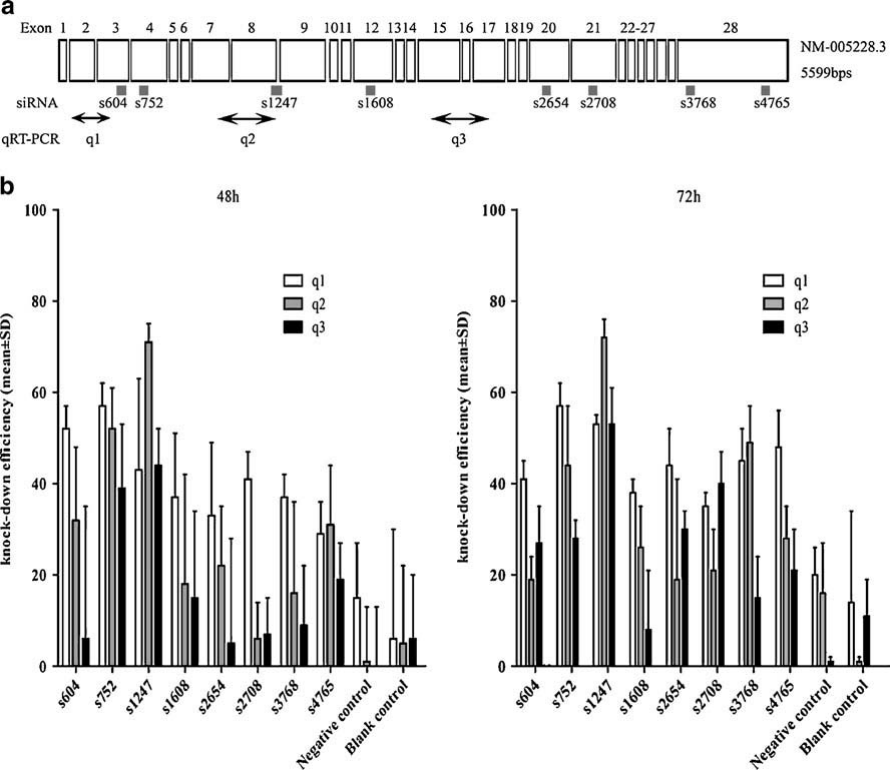
**Determination of EGFR siRNA knockdown efficiency in H358 cells with RT-qPCR**. (A) Schematic diagram of the EGFR gene exon (boxes) structure and location of siRNAs and RT-qPCR primer sets. For the siRNAs sequences, see Table 1. For the primers sequences, see Table 2. Primer sets were named according to the order of the sequences. (B) EGFR mRNA knockdown efficiency detected by RT-qPCR amplification from EGFR siRNA-treated H358 cells using either the primer set q1,q2 and q3 at 48 hrs (Left), and at 72 hrs (Right). Transfections were performed in triplicate. The percentages are relative to the mock treated control.

In the present study, we evaluated the RT-qPCR assay used to measure the siRNA knockdown of EGFR expression. In our experiments, we were interested in determining the ability of a series of EGFR siRNAs to knock down the levels of endogenous EGFR mRNA expression in a human lung cancer cell line expressing wild type EGFR. To exclude effects of transfection efficiency on siRNA efficacy, we determined H358 cells transfection efficiency with two approaches: cell fluorescence by siGLO Transfection Indicators and cell death induced by TOX Transfection Control. The transfection efficiency was higher than 81% at 48 hrs and 92% at 72 hrs, as accessed by either CellTiter-Blue^® ^Cell Viability Assay, or fluorescence, as assessed by fluorescence microscopy (data not shown). These data suggested that the transfection efficiency was nearly optimal with all siRNAs tested. Of the eight siRNAs examined, s604, s752 and s1247 siRNAs showed the highest efficiency in the knockdown of the EGFR mRNA (Figure 1B). With primer sets q1 and q3 initially used, the reduction of EGFR transcript levels was not greater than 57% at 48 hrs. We then redesigned a primer set q2 to overlap or encompass the EGFR target site of siRNA s1247. Upon reexamination with primer set q2, which encompasses the target site and using siRNA s1247, a 71% decrease of the EGFR mRNA level was observed. It was the strongest effect observed for all eight siRNAs tested. In contrast, the primer set q2 underestimated the knockdown efficacy in the siRNA s604 and s752 experiments in comparison to what was measured with primer set q1 for the same siRNAs. This primer set q1 amplifies a sequence that locates nearer to the target sequence of s604 and s752. Similar results were observed at 72 hrs: with primer set q2, a knockdown of 72% was found in the s1247 experiments, whereas with the same siRNA q1 detected a 53% knockdown and q3 a 53% knockdown (Figure 1B).

RT-qPCR amplification of a specific targeted mRNA sequence reflects the integrity of that fragment, but does not necessarily represent an intact mRNA. Our data suggest that the EGFR mRNA fragments amplified by the q1 or q3 primer sets after transfection of s1247, remain more intact despite RNA cleavage and thus result in an overestimation of the amount of remaining intact target mRNA. The relatively higher knockdown read-out with primer set q1, after transfection with s604 and s752, compared to q3, is also consistent with this observation (Figure 1B). These results thus suggest that s1247 is the most effective of the siRNAs tested.

**Figure 2:**
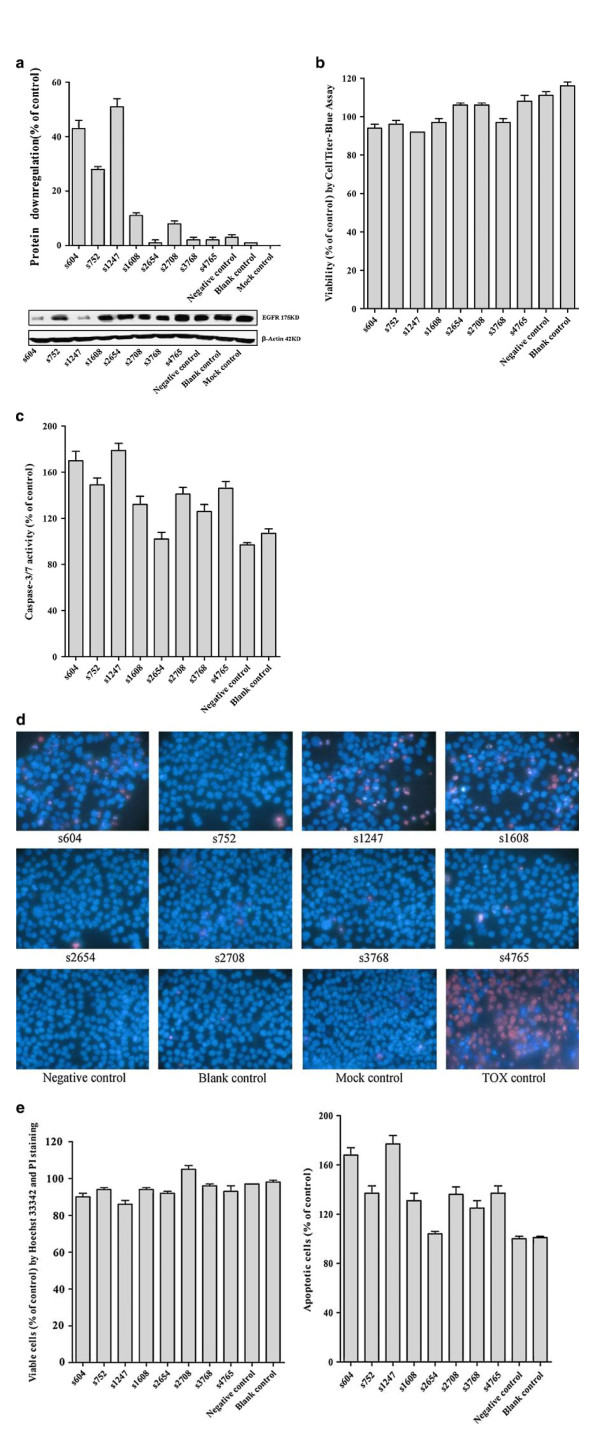
**Down-regulation of EGFR protein levels and phenotypic consequences of EGFR knockdown**. (A) Western blot was performed to determine the down-regulation levels of EGFR protein. (B) Cell viability was detected by CellTiter-Blue^® ^Cell Viability Assay. (C) Caspase-3/7 activity was measured by Apo-ONE^® ^Homogeneous Caspase-3/7 Assay. (D, E) Viable and apoptotic cells were counted with Hoechst 33342 and propidium iodide (PI) double fluorescent chromatin staining. The aggregate results are consistent with the mRNA knockdown results obtained in the RT-qPCR experiments and confirm that of the siRNAs tested, the s1247 is the most powerful siRNA on H358 cells to down-regulate the EGFR protein level, inhibit the cell viability and induce apoptosis in comparison to other siRNAs (Results see Table 1). Idem as above.

To further corroborate this hypothesis we analyzed EGFR protein levels with western blot and studied possible phenotypic consequences of EGFR down-regulation in H358 cells. Cell viability and caspase-3/7 activity were measured and, in addition, we evaluated the induction of apoptosis. The aggregate results of these experiments are consistent with the mRNA knock down results obtained in the RT-qPCR experiments and confirm that of the siRNAs tested, the s1247 is the most powerful siRNA on H358 cells to down-regulate the EGFR protein level, inhibit the cell viability and induce apoptosis in comparison to other siRNAs (Table 1, Figure 2).

**Table 1:** Phenotypic consequence of EGFR downregulation by siRNAs

Name of siRNA	exon	sequence	**Location**^**a**^	Designed by	RNA knockdown Measured with q2 (%)	Protein downregulation (%)	Viability (%)	Caspase-3/7 (%)	Apoptotic cells (%)	Viable cells (%)
EGFR siRNA604	3	GCAGTCTTATCTAACTATGATGCAA	C.604_628	Invitrogen	19 ± 5	43 ± 3	94 ± 2	170 ± 8	168 ± 6	90 ± 2
EGFR siRNA752	4	GCAGTGACTTTCTCAGCAA	C.752_770	Eurogentec	44 ± 13	28 ± 1	96 ± 2	149 ± 6	137 ± 6	94 ± 1
EGFR siRNA1247	8-9	GCAAAGTGTGTAACGGAATAGGTAT	C.1247_1271	Invitrogen	72 ± 4	51 ± 3	92 ± 0	179 ± 6	177 ± 7	86 ± 2
EGFR siRNA1608	12	GGAGATAAGTGATGGAGAT	C.1608_1626	Eurogentec	25 ± 9	11 ± 1	97 ± 2	132 ± 7	131 ± 6	94 ± 1
EGFR siRNA2654	20	GGGAACACAAAGACAATAT	C.2654_2672	Dharmacon	18 ± 22	1 ± 1	106 ± 1	102 ± 6	104 ± 2	92 ± 1
EGFR siRNA2708	20-21	TCGCAAAGGGCATGAACTA	C.2708_2726	Dharmacon	21 ± 9	8 ± 1	106 ± 1	141 ± 6	136 ± 6	105 ± 2
EGFR siRNA3768	28	GGACTTCTTTCCCAAGGAA	C.3768_3786	Eurogentec	48 ± 8	2 ± 1	97 ± 2	126 ± 6	125 ± 6	96 ± 1
EGFR siRNA4765	28	AGAATGTGGAATACCTAAGG	C.4766_4785	*	2 ± 7	2 ± 1	108 ± 3	146 ± 6	137 ± 6	93 ± 3
Negative siRNA		proprietary sequence designed by Eurogentec			16 ± 11	3 ± 1	111 ± 2	97 ± 2	100 ± 2	97 ± 0
Blank control					1 ± 5	1 ± 0	116 ± 2	107 ± 4	101 ± 1	98 ± 1

^a^Reference sequence identical to NM_005228.3 ^b ^Modified from [7].

To verify whether the effect of primer set choice on RT-qPCR results is also observed with other messengers, we transfected a positive, validated GADPH siRNA (Invitrogen, proprietary sequence) into H358 cells and measured the GAPDH mRNA knockdown level with three different primer sets located in different positions. With different locations, the primers detected different knockdown efficiencies (data not shown), and the best primer set was GAPDH 820F and 1106R (Table 2). The results from GAPDH RNAi are analogous to the data obtained with the EGFR mRNA suggesting that these findings can be generalized, i.e., measurements of the knockdown efficiency can be influenced by the primer set used for RT-qPCR.

**Table 2:** Primer Sequences for EGFR and GAPDH Transcripts used for Real-Time Quantitative reverse Transcriptase Polymerase Chain Reaction

Name	Forward	exon	Sequence	**Location**^**a**^	**Reverse**^**b**^	exon	Sequence	LocationΔ	Length(bp)
q1	EGFR 370F	2	GGCACTTTTGAAGATCATTTTCTC	c.370_393	EGFR 514R	3	CTGTGTTGAGGGCAATGAG	c.514_532	163
q2	EGFR 1089F	7	CGAGGGCAAATACAGCTT	c.1089_1106	EGFR 1263R	9	AAATTCACCAATACCTATT	c.1263_1281	193
q3	EGFR 2034F	15	GGCAGGAGTCATGGGAGAA	c.2034_2052	EGFR 2168R	17	GCGATGGACGGGATCTTAG	c.2168_2186	153
	GAPDH 240F	3	TTGCCATCAATGACCCCTTCA	c.240_260	GAPDH 395R	5	CGCCCCACTTGATTTTGGA	c.395_413	173
GAPDH	GAPDH 820F	8	TGAACGGGAAGCTCACTGG	c.820_837	GAPDH 1106R	9	TCCACCACCCTGTTGCTGTA	c.1106_1125	306
	GAPDH 1016F	8	ACCCACTCCTCCACCTTTG	c.1016_1034	GAPDH 1175R	9	CTCTTGTGCTCTTGCTGGG	c.1175_1193	177

^a^Reference sequence: EGFR NM 005228.3, GAPDH NM 002046.3

^b^The reverse primers were also used to initiate cDNA synthesis.

SiRNA induced cleavage of mRNA appears to be followed by degradation of the messenger, which is assumed to be complete 24-48 hrs after siRNA treatment [4]. Consequently, the position of the PCR primers within the mRNA (cDNA) is generally considered irrelevant, and many publications do not provide a rationale for the primers chosen and specifically do not include qPCR primers that overlap with the siRNA target sequence. However, there are indications that mRNAs are incompletely degraded 24 hrs after transfection as evidenced by others in a Northern blot analysis following treatment with siRNAs against coagulation factor III [2]. Our results corroborate and strengthen observations made by Shepard et al. [3]. In a study targeting the connective tissue growth factor in human trabecular meshwork cells, they found that the location of the qPCR primers relative to the siRNA target sequence profoundly influenced the RT-qPCR results, by as much as 60%, 24 hrs post-transfection. They also showed that these results were independent of siRNA concentration, siRNA:lipid ratio, or transfection efficiency. Primer sets that did not overlap the siRNA target sequence yielded a lower knockdown efficiency, which can be explained by the existence of partially degraded mRNA molecules that are still detected in the RT-qPCR assay. However these results favoring a primer position effect were based upon analysis 24 hrs post treatment, a time point at which abundant mRNA may have been cleaved by the argonaute protein, but not yet degraded by exonucleases. More recently, the importance of primer position for assessment of MYCN oncogene siRNA silencing in a human neuroblastoma cell line (IMR-32) was also described by the group of Vandesompele [5]. Their RT-qPCR analysis was at 48 h post transfection. Our present results strongly suggest that amplification of incompletely degraded mRNA molecules, as observed with the EGFR q1 and q3 primer sets with siRNA s1247, may lead to underestimation of the siRNA efficacy, at least up to 72 hrs post-treatment. Nevertheless, the fact that these position effects were measureable in different cell types (trabecular cells, neuroblastoma and lung cancer cell lines), and with different genes (connective tissue growth factor, MYCN oncogene, EGFR, GAPDH), strongly suggests that incomplete degradation of mRNA following siRNA treatment is a general phenomenon that may significantly affect RT-qPCR results in RNAi research.

## Conclusions

We thus conclude that there is an unexpected but significant interdependence between the EGFR targeting siRNA sequence and the RT-qPCR amplification region for assessing the efficacy of the siRNA target gene knockdown and that this finding can be extended to other mRNAs in lung cancer cells. We therefore recommend that qPCR primers for siRNA work should span the putative siRNA cleavage site, thus avoiding the amplification of mRNA molecules that underwent only the initial steps of the siRNA induced degradation process. It is also recommendable to test more than one primer set at different locations on the mRNA, particularly for mRNA targets that appear to be refractory to siRNA-mediated cleavage. Ideally, at least one of the primer sets should amplify a region encompassing the siRNA recognition sequence to ensure optimum siRNA efficacy readout.

## Methods

### siRNA transfection

Eight siRNAs targeting wild type EGFR sequences were analyzed (Table 1, Figure 1A). EGFR siRNA604 and EGFR siRNA1247 were from Invitrogen (*Ref. SKU#12938-076, EGFR validated stealth duo pack, Invitrogen, Merelbeke, Belgium*); the other siRNAs were synthesized by Eurogentec (*Eurogentec S.A., Liege, Belgium*). All the sequences used for siRNAs were finally blasted http://blast.ncbi.nlm.nih.gov/Blast.cgi to avoid silencing unrelated genes. The glyceraldehyde-3-phosphate dehydrogenase (GAPDH) positive control siRNA was from Invitrogen (*ref. SKU#12935-140 Stealth RNAi GAPDH Positive Control, Invitrogen Merelbeke, Belgium*). Eurogentec provided the negative control siRNA *(OR-0030-neg 05, Eurogentec S.A., Liege, Belgium)*, a proprietary siRNA sequence that does not correspond to any human gene. TOX Transfection Control and siGLO Green Transfection Indicators were ordered from Thermo Scientific Dharmacon (*ref. D-001630-01-02; D-001500-01-05*, *Thermo Scientific Dharmacon, Blenheim, England*). The human non-small cell lung cancer (NSCLC) cell line NCI-H358, wild type for EGFR, was obtained from the American Type Culture Collection (*ATCC, Netherlands*) and cultured in RPMI 1640 medium (*Invitrogen Corp., Gent, Belgium*), supplemented with 10% heat-inactivated fetal bovine serum (*Perbio Science NV, Erembodegem, Belgium*), 2 mM L-glutamine and 1 mM sodium pyruvate at 37°C in a humidified incubator with 5% CO_2 _but without penicillin or streptomycin, which will otherwise cause cell death during lipofectamine transfection. Cell line H358 was transfected with EGFR siRNAs, a negative control siRNA or positive GAPDH control siRNA at 40 nM using 1.5 μl Lipofectamine 2000 (*Invitrogen Merelbeke, Belgium*) per well in a 24-well format in triplicate.

### RNA extraction and LightCycler real time PCR

The EGFR mRNA level was determined 48 hrs and 72 hrs post-transfection by RT-qPCR. Total cellular RNA isolation was performed on the ABI PRISM 6100 prepstation using the AbsoluteRNA Solution (*Applied Biosystems, Lennik, Belgium*) to remove contaminating DNA and PCR inhibitory substances. RNA concentrations were determined using a ND-1000 NanoDrop (*Thermo Fisher Scientific, Wilmington, Delaware USA*), and used for normalization of the input RNA in the RT-qPCR. Two hundred nanogram of cellular RNA was converted to cDNA with a specific reverse primer (sequences see Table 2) using Moloney Murine Leukemia Virus (MMLV) Reverse Transcriptase (*RT-RTCK-03, Eurogentec S.A., Liege, Belgium*) in a total volume of 10 μl. The reverse transcription step was performed on an Applied Biosystems Thermocycler at 25°C for 10 minutes for the initial step, 30 minutes at 48°C for the reverse transcription step and 95°C for 5 minutes to inactivate the reverse transcription enzyme. Oligonucleotide primers (*purchased from Eurogentec S.A., Liege, Belgium*) for EGFR were based upon GenBank sequence (*ENSEMBL sequence: ENST00000275493, identical to NM_005228.3*) and for GAPDH, *Ensembl sequence: ENSG00000111640, identical to NM_002046.3 *). The primers were designed using the Roche LightCycler Probe Design Software v1.0 (*Idaho Technology, Salt Lake City, UT*), FastPCR (Version 5.4 for Windows), followed by BLAST analysis http://blast.ncbi.nlm.nih.gov/Blast.cgi. Several transcript-specific primer sets were designed to be located in different exons or spanning an exon-exon border to avoid amplification of genomic DNA (Table 2). RT-qPCR analysis was carried out on a LightCycler 1.5 using the Faststart DNA master SYBR green mastermix, and with three different primer sets (Table 2, Figure 1A). The quality of the amplified fragments was monitored using melting curve analysis and agarose-gel-electrophoresis. Quantitative values were obtained from the PCR quantification cycle number (Cq) at which point the increase in signal for the PCR product was exponential. The target mRNA abundance in each sample was normalized to its GAPDH mRNA level as ΔCq = Cq_EGFR_-Cq_GAPDH_. The value ΔΔCq was defined as the difference with a mock tranfected control. Experiments were performed in triplicate. The knock-down ratio of EGFR RNA expression was calculated with the formula: (1-1/2^ΔΔCq^) × 100% [6].

### Protein, viability and apoptosis experiments

Protein down regulation was analyzed by western blot with a primary antibody against EGFR (1:1000 dilution, *Anti-EGFR, non-phospho-Tyr1173, clone 20G3, Millipore mouse monoclonal IgG*_*1К*_*, Catalog # 05-484, Lot # 30267, Bio-connect, Huissen, The Netherlands*). The inhibition ratio of EGFR protein expression was calculated with the following formula: inhibition ratio of EGFR protein expression = (1 - the relative intensity of EGFR expression in the siRNA experiment group/the relative intensity of EGFR in the mock control group) × 100. Cell viability was determined with the CellTiter-Blue^® ^Cell Viability Assay (*G8080, Promega, Madison, USA*). The calculation of results was as: fluorescence of siRNA/mock control × 100. Caspase-3/7 activity was measured by Apo-ONE^® ^Homogeneous Caspase-3/7 Assay (*G7790, Promega, Madison, USA*). Caspase-3/7 activity was measured by fluorescence: assay siRNA/mock control × 100. The effects of EGFR siRNAs on apoptosis and nuclear morphology in the cells were assessed by Hoechst 33342 (*Sigma-Aldrich N.V. Bornem, Belgium*) and propidium iodide (PI, *Sigma-Aldrich N.V. Bornem, Belgium*) double fluorescent chromatin staining. Viable, apoptotic and necrotic cells were counted in 10 different fields under a 200 × magnification in each well in three independent experiments by two persons, and the average result was compared to the mock control. Values were presented as the mean ± standard deviation (SD).

## Competing interests

The authors declare that they have no competing interests.

## Authors' contributions

GC carried out the primer and siRNA designing, transfections of the siRNA, RNA extraction, real time PCR and protein, viability, apoptosis experiments, and drafted the manuscript. PK conceived of the study, carried out primer and siRNA designing, supervision of all the experiments, and helped to draft the manuscript. ET participated in the design of the study and interpretation of data, and helped to revise the manuscript. JDG participated in the design of the study and coordination and helped to revise the manuscript. All authors read and approved the final manuscript.
